# Prevalence of COVID-19 Vaccine Hesitancy Among Healthcare Workers in Nigeria: A Systematic Review and Meta-Analysis

**DOI:** 10.3389/ijph.2025.1607655

**Published:** 2025-02-05

**Authors:** Taagbara Jolly Abaate, Dabota Yvonne Buowari, Utchay A. Agiri, Tamunodiepiriye Inimgba, Vivian Ifeoma Ogbonna, Chizaram Onyeaghala, Glory Ovunda Worgu, Abiye Somiari, Emmanuella I. Ezebuiro, Ibe Arthur Onuah

**Affiliations:** ^1^ Department of Community Medicine, University of Port Harcourt Teaching Hospital Nigeria, Port Harcourt, Nigeria; ^2^ Department of Accident and Emergency Medicine, University of Port Harcourt Teaching Hospital, Port Harcourt, Nigeria; ^3^ Department of Family Medicine, University of Port Harcourt Teaching Hospital, Port Harcourt, Nigeria; ^4^ Department of Internal Medicine, University of Port Harcourt Teaching Hospital, Port Harcourt, Nigeria; ^5^ Department of Obstetrics and Gynaecology, University of Port Harcourt Teaching Hospital, Port Harcourt, Nigeria; ^6^ Department of Surgery, University of Port Harcourt Teaching Hospital, Port Harcourt, Nigeria

**Keywords:** COVID-19, vaccine hesitancy, non-acceptance, healthcare workers, Nigeria

## Abstract

**Objective:**

The purpose of this systematic review and meta-analysis was to determine the prevalence of COVID-19 vaccine hesitancy among Nigerian healthcare professionals.

**Methods:**

An extensive language-unrestricted literature search was conducted across PubMed, Scopus, the Cochrane Library, and the African Index Medicus to identify studies reporting hesitancy to COVID-19 vaccines among healthcare workers in Nigeria. Quality assessment was performed using the Newcastle-Ottawa scale for cross-sectional studies. A single-arm meta-analysis was performed using a random-effects model.

**Results:**

Of the 206 articles, 22 publications with 20,724 participants were included. The pooled prevalence of COVID-19 vaccine hesitancy was 75% (95% CI: 61%–88%, I^2^ = 99.69%, P < 0.001). Reasons for hesitancy, including concerns about side effects, lack of trust, and safety, were prevalent at 76% (CI: 0.57–0.94, I^2^ = 99.24%, P < 0.001), 55% (CI: 0.042–0.272, I^2^ = 97.42%, P < 0.001), and 68% (CI: 0.047–0.89, I^2^ = 98.59%, P < 0.001), respectively.

**Conclusion:**

There was significant hesitancy among Nigerian healthcare workers towards COVID-19 vaccination; thus, strategies to increase vaccination acceptance among healthcare workers should be developed.

## Introduction

Viral diseases present considerable challenges to public health, often spreading rapidly across borders and proving difficult to contain. The COVID-19 pandemic, caused by the SARS-CoV-2 virus and declared in 2020, highlighted the interconnectedness of our global society [1, 2]. The virus rapidly crossed international borders, tested healthcare systems, and revealed our vulnerability to unpredictability in nature [3]. Despite the tragedy, the event sparked phenomenal scientific collaboration and inventiveness, emphasising the importance of planning and international cooperation [4]. The global impact of the COVID-19 pandemic, which has resulted in increased rates of illness and death, has prompted a reevaluation of one-health principles and underscored the critical importance of having a resilient public health infrastructure [5–7]. The World Health Organisation (WHO) estimated that approximately 83 million SARS-CoV-2 infections had been recorded, with 1.9 million of these infections occurring in Africa as of January 2021, with most cases occurring in Kenya, South Africa, Algeria, Ethiopia, and Nigeria [7]. Therefore, the implementation of cost-effective measures like vaccination in all countries is a crucial step in the fight against the virus.

Vaccination is a valid tool for containing diseases that can easily spread from one person to another. However, it is common for individuals to refuse vaccine inoculation. As of 19 November 2021, the proportion of unvaccinated people has climbed to 97.15% of the total population, a figure that is particularly concerning given the current hesitation among the healthcare workers [8, 9]. Vaccine hesitancy is influenced by various context-specific factors that vary over time, region, and vaccine type [10–14]. Considerations of convenience, confidence, complacency, and sociodemographic and cultural factors play a role in deciding whether or not to take a vaccine [7]. Lack of acceptability and disinformation poses challenges to comprehensive vaccine coverage and community immunity.

In Nigeria, similar to many other countries, global best practises such as handwashing, mask wearing, social distancing, and vaccination campaigns were adopted to curb COVID-19 transmission. The vaccine rollout strategy involved phased distribution, prioritising healthcare workers and then the general population, following the reception of approved vaccines in batches [15].

These health workers are on the frontlines meeting and caring for many patients with confirmed COVID-19 and undiagnosed people with symptoms prevalent amongst coronavirus infected individuals [16, 17]. However, uptake among frontline workers is low despite the availability of vaccines [14].

Vaccine hesitancy is caused by a delay in accepting or declining vaccines despite vaccine availability [18]. When this occurs among healthcare personnel, it represents a hurdle to attaining global immunisation targets. This is because health workers’ vaccination uptake affects the community acceptability of COVID-19 vaccines [11]. A core duty of health professionals is to educate the public about disease prevention and control, and their attitudes may shape the general public’s perception of the impact of an intervention (COVID-19 vaccine) on reducing the burden of the pandemic [18].

According to Olu-Abiodun et al., in October 2020, the acceptance rate for healthcare professionals was 55.5%; it dropped to 32.5% in January 2021; and reached 45.6% in March 2021 [7]. Although these levels vary across regions, acceptance rates remain problematic. There is a preponderance of studies on the prevalence of COVID-19 vaccination hesitancy among healthcare workers in Nigeria, but there is a paucity of literature with pooled estimates, which can help guide policies and strategies in the current or future pandemics on the acceptability of vaccines and other interventions targeted at protecting the health workforce. If Nigeria is to achieve herd immunity to COVID-19 infection, then understanding the factors that drive COVID-19 vaccine hesitancy among such an important population and addressing them accordingly is key because healthcare workers play an important role in disease prevention and can drive positive COVID-19 vaccination attitudes and practices, not just in hospitals but in communities [17].

It is crucial at this point, therefore, to conduct a systematic review and meta-analysis to obtain pooled estimates from the literature on the current level of COVID-19 vaccine hesitancy among healthcare workers in Nigeria, especially at this time when vaccines are available and accessible, and the pandemic is over.

### Aims and Objectives

This study primarily aims to determine the prevalence of COVID-19 vaccine hesitancy among healthcare providers in Nigeria. The secondary objective of this review was to identify the factors contributing to COVID-19 vaccine refusal among Nigerian healthcare workers.

### Research Question

The research question was; what is the prevalence of COVID-19 vaccine hesitancy among Nigerian healthcare workers?

## Methods

### Eligibility Criteria

This review included observational studies involving healthcare providers from various states in Nigeria who refused COVID-19 vaccination. These providers work in healthcare facilities and deliver care directly (e.g., doctors, nurses) or indirectly (e.g., pharmacists, laboratory scientists) [19]. Publications focusing on medical students, hospital administrative staff, non-observational studies on healthcare workers, animal studies, and studies on the general population were excluded. Healthcare workers represent a heterogeneous population; therefore, studies that were similar in terms of the participants were grouped for analysis.

### Information Sources

The study followed the Preferred Reporting Items for Systematic Reviews and Meta-Analyses (PRISMA) guidelines [20] and is registered in the PROSPERO database (registration number CRD42022365489). The review protocol is available in Ibom Medical Journal at http://dx.doi.org/10.61386/imj.v16i2.305. A comprehensive literature search was conducted across four major databases: PubMed, Cochrane Library, Scopus (via Publish or Perish software), and the African Index Medicus [21]. The search terms were based on the condition under study (COVID-19 vaccine hesitancy): context (Nigeria) and population of interest (Healthcare workers) to retrieve relevant articles published from March 1st, 2021, to March 27, 2022, without language restriction. In response to the COVID-19 pandemic, Nigeria received its first shipment of vaccines from the COVID-19 Vaccines Global Access Facility (COVAX) on March 2, 2021 [7]. The country implemented a four-phase National Deployment and Vaccination Plan (NDVP), with the first phase prioritizing healthcare workers and other frontline personnel [22]. The authors anticipated that reports on vaccine non-acceptance would surface within a year of the vaccine’s introduction and applied a date filter, though the review extended beyond 1 year. However, all articles found during this period were written in English, with no available translations. The search string applied in PubMed is as follows: (COVID-19 OR COVID-19 OR Coronavirus OR COVID OR SARS-CoV-2 OR sars-cove-2) AND (Vaccination OR vaccine OR vaccine* OR immunisation OR immunisation) AND (“healthcare workers” OR “health personnel” OR physician OR nurse OR doctor OR residents OR pharmacist OR “laboratory scientist” OR “lab technician”) AND (Rejection OR hesitancy OR compliance OR attitude OR refusal OR non-acceptability) AND Nigeria. Records found were reviewed to determine whether they met the inclusion criteria. We also performed a hand search for grey literature, conference abstract proceedings, reference lists of the included publications, and citations in Google Scholar.

### Screening and Selection Process

**FIGURE 1:**
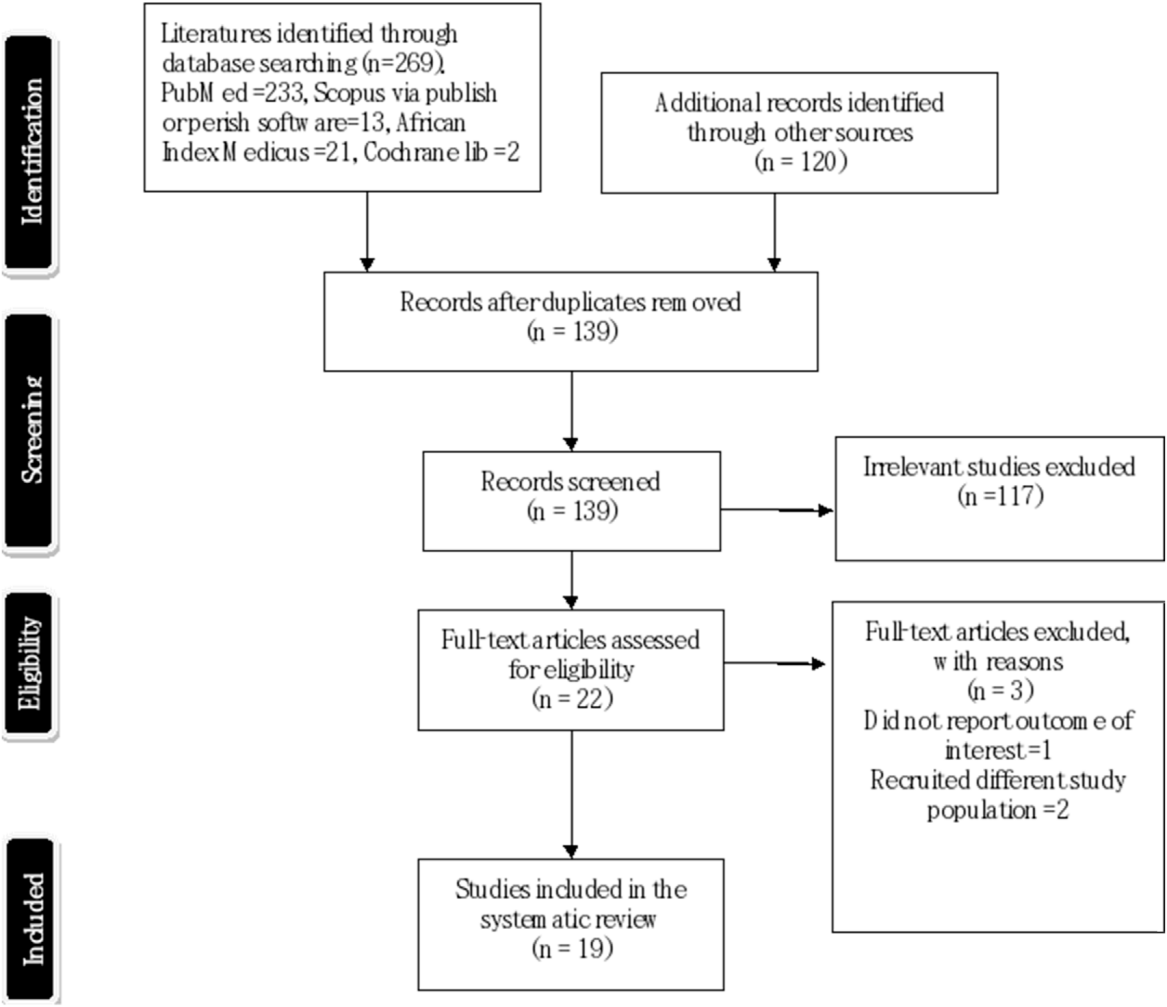
Preferred Reporting Items for Systematic Review and Meta-analysis flow diagram showing process of selection, inclusion and exclusion of studies reviewed in 2021/2022 (Nigeria 2021/2022).

Three independent co-authors (CO, UA, and TA) screened the title and abstract, followed by a full-text screening of the articles identified based on predefined eligibility criteria. Studies were included if they assessed the prevalence of COVID-19 vaccine hesitancy among healthcare providers (medical doctors, public health officers, chemists, medical laboratory scientists, nurses, and others) [21, 23, 24] through observational designs in any of the 36 states of Nigeria plus the federal capital territory [22]. Of the 389 articles identified, 250 duplicate items were removed and 117 were eliminated after title and abstract screening. We excluded studies unrelated to COVID-19 vaccine hesitancy, studies on general populations or non-observational designs, and those conducted outside Nigeria, resulting in 22 abstracts for full-text review. Three articles were excluded during full-text screening (see Figure 1; PRISMA flow diagram). Author disagreements were resolved through discussion.

### Risk of Bias Assessment

Quality assessment utilized the adapted Newcastle–Ottawa Scale for cross-sectional studies [25]. This scale is graded on a 10-point scale and consists of three domains. Domain 1 evaluates the methodological quality of each study (with a maximum of 5 stars), domain 2 assesses the comparability of the studies (with a maximum of 2 stars), and domain 3 evaluates the outcome measures and related statistical analyses (with a maximum of 3 stars.) [25]. Furthermore, the review categorised the overall quality of the studies into three groups: low risk of bias (scored 7–10), moderate risk of bias (scored 5–6), and high risk of bias (scored 0–4). This assessment process was conducted by three independent co-authors (EE, TA, and OI), and the final score for each study was determined by averaging their assessments. Any discrepancies that arose during this process were resolved through discussion. See Supplementary Material S1.

### Data Extraction

Data were independently extracted by three co-authors (OI, WO, and TA), capturing authorship, publication year, research location, study design, population, and sample size. The main outcome measures (proportion of vaccine hesitancy) and details related to the secondary objectives of the review (factors contributing to COVID-19 vaccine hesitancy) were also abstracted. For studies that reported the outcome of interest (vaccine hesitancy) in percentages along with sample sizes [26–31], we manually computed the absolute prevalence rate. In one study with missing data on the main outcome [12], we attempted to contact one of the study authors but did not receive a response. Despite this, the study was still included in the qualitative synthesis. Data extraction was performed using Microsoft Office Excel, which was prepared by the team. To ensure the appropriateness of the Excel sheet, we piloted it using four studies. In cases of disagreement, resolution was achieved through discussion.

### Data Analysis

A single-arm meta-analysis with a random-effects model was used to estimate the pooled prevalence of COVID-19 vaccine hesitancy among healthcare providers in Nigeria at a 95% confidence interval presented in a forest plot. The relative weight of each study and the prediction interval are also presented. Separate estimations were made for the proportions of COVID-19 vaccine hesitancy and factors influencing vaccine uptake. Factors contributing to COVID-19 vaccine hesitancy were subjected to meta-analysis if they had been assessed and data from at least two studies were available. To enhance variance stability, the proportions were transformed using the Freeman–Tukey Double Arcsine Transformation method [32, 33]. The assessment of heterogeneity was performed using the Cochrane Q statistic to determine data variability and its statistical significance from naught, Tau, and Tau^2^ evaluated by analysing the variance in effect size measurement and the inconsistency index (I^2^) [32, 33]. The I^2^ statistic was interpreted based on Higgins and Thompson’s classification, in which percentages of 25%, 50%, and 75% were considered indicative of low, moderate, and high heterogeneity, respectively [32]. Funnel plot asymmetry, Egger’s regression, and Begg’s rank correlation tests were used to assess publication bias. Subgroup analysis was performed on studies that recruited a cadre of HCWs (medical doctors). All statistical analysis were carried out in JAMOVI 2023 version 2.4.5 [34].

## Results

Among the 389 articles retrieved, 139 remained after removing duplicates. Following title and abstract screening, 177 articles were excluded as irrelevant to the study. We evaluated 22 full-text papers for eligibility, and three articles were excluded from the final data synthesis. Consequently, 19 studies were analysed. See Figure 1 (PRISMA flow diagram) shows the process of selection, inclusion and exclusion of studies.

### Characteristics of the Included Studies

The articles included in this study were published in 2021 (n = 6) and in 2022 (n = 13). The studies involved 20,724 participants and were conducted across six geopolitical zones and states in Nigeria and published in English. All studies employed a cross-sectional design with physicians, nurses, medical laboratory scientists, pharmacists, physiotherapists, radiographers, optometrists, community health workers, and dental technicians as study participants. The majority of them (79%) were assessed to have low quality but were included in the review. An overview of the included studies is presented in Table 1.

**TABLE 1:** Characteristics of the studies included in this systematic review and meta-analysis for the period 2021/2022 (Nigeria, 2021/2022).

Author (Year)	Title of study	Country of study	Study location	Study design	Study population	Sample size	Prevalence of COVID-19 vaccination hesitancy event %		Risk of bias
[35]	COVID-19 vaccine hesitancy among healthcare workers: Assessment of its magnitude and determinants during the initial phase of national vaccine deployment in Nigeria	Nigeria	All states in Nigeria	Cross-sectional	Nurse/midwife, CHEW, doctors, environmental health assistants, Laboratory Scientist, Laboratory technologist, optometrist, pharmacists, pharmacy technologist	10,184	858	8.00	Moderate
[12]	Vaccine hesitancy: Pattern of side effects of the first dose of AstraZeneca COVID-19 vaccine among healthcare workers in Enugu	Nigeria	Enugu state	Cross-sectional	Healthcare workers	89	N/A		High
[36]	COVID-19 Vaccine Knowledge and Acceptability among Healthcare Providers in Nigeria	Nigeria	The six geopolitical regions	Cross-sectional	Doctor, Nurse, Radiographer/Imaging Scientist, Public Health Workers, Dentist/Dental Therapist, Optometrist, Scientific Officer, Medical Laboratory Scientist, Pharmacist, Medical Record Officer, Physiotherapist and Hospital Cleaner	445	207	46.50	High
[13]	COVID-19 Vaccine Hesitancy and Determinants of Acceptance among Healthcare Workers, Academics and Tertiary Students in Nigeria	Nigeria	All states in Nigeria	Cross-sectional	Healthcare workers	1,525	1,079	70.75	High
[37]	Pharmacists’ readiness to receive, recommend and administer COVID-19 vaccines in an African country: an online multiple-practice settings survey in Nigeria	Nigeria		Cross-sectional	Hospital and community Pharmacists	509	166	32.70	High
[26]	Predictors of COVID-19 Vaccine Acceptance among Nigerian Medical Doctors	Nigeria		Cross-sectional	House officers, medical officers, private practitioner, resident doctors, consultants, Lecturers and unemployed doctors	830	220	26.50	Moderate
[27]	Acceptance of COVID-19 Vaccines among Healthcare Workers in Lokoja, Nigeria	Nigeria	Lokoja	Cross-sectional	Medical doctor, nurse, pharmacist, administrative staff, Account, Attendant and Laboratory staff	840	425	88.60	High
[28]	COVID-19 vaccine hesitancy among healthcare workers and its sociodemographic determinants in Abia State, Southeastern Nigeria: a cross-sectional study	Nigeria	Abia state	Cross-sectional	Doctor, Nurse, allied professions, and non-clinical staff	422	213	50.50	High
[14]	Willingness to Accept COVID-19 Vaccine among Anesthetists in Nigeria	Nigeria		Cross-sectional	Anesthetists	195	92	47.20	High
[29]	Prevalence and Predictors of COVID-19 Vaccine Hesitancy among Healthcare Workers in Tertiary Healthcare Institutions in a Developing Country: A Cross-Sectional Analytical Study	Nigeria	Imo state	Cross-sectional	Doctor, nurse/midwife, Pharmacists, Laboratory scientist, and others	347	123	35.40	High
[38]	Assessment of Knowledge and Acceptance of COVID-19 Vaccinations among Healthcare Workers in Kano State, Nigeria	Nigeria	Kano State	Cross-sectional	Medical doctors and nurses, and midwives	864	223	32.70	High
[39]	COVID-19 vaccine hesitancy among health workers in surgical departments in Port Harcourt, Nigeria	Nigeria	Port Harcourt	Cross-sectional	Student (Undergraduate), Consultants, Resident Doctor, Medical officer, pharmacist, Nurse, Medical Laboratory Scientist, Physiotherapist, Administrative Staff and	302	141	47.70	High
[40]	COVID-19 Vaccine Hesitancy among Medical Doctors at a Tertiary Healthcare Facility in the Niger-Delta, Nigeria	Nigeria	Niger Delta (Bayelsa state)	Cross-sectional	Medical doctors	102	72	70.60	Moderate
[41]	Perceptions of the COVID-19 vaccine and willingness to receive the vaccine among health workers in Nigeria	Nigeria	Ondo State Edo State Delta State	Cross-sectional	Doctor, Nurse, Medical laboratory scientist/technician, pharmacist/physiotherapist, other health workers (administrator, health attendant)	1,470	654	44.50	High
[30]	Knowledge, acceptance, and Hesitancy of COVID-19 Vaccine among healthcare workers in Nigeria	Nigeria		Cross-sectional	Ancillary Support Staff, Dental Technicians, General Medical Practitioners, House Officers, Lab Scientists, Medical Consultants, Nurses/Midwives, Optometrists, Pharmacy, Physiotherapists, PHC, Radiographer, Resident Doctor	1,094	500	39.68	High
[42]	COVID-19 vaccination hesitancy among Healthcare workers in Kaduna State, Nigeria	Nigeria	Kaduna State	Cross-sectional	Community Health Officers, Laboratory scientists, medical doctors, nurses, pharmacists, physiotherapists, and others	351	183	52.10	High
[43]	Drivers of COVID-19 Vaccine Uptake among Healthcare Workers (HCWs) in Nigeria	Nigeria		Cross-sectional	Physician, nurse of midwife, Community health worker Other public health practitioner, Pharmacist, PPMV, Laboratory staff	496	164	31.00	High
[44]	Predictors of COVID-19 Vaccine Acceptance among Healthcare Workers in Nigeria	Nigeria	Northern, Western and Eastern regions of Nigeria	Cross-sectional	Physicians, nurses, pharmacist, physiotherapist, radiographers and, scientist	710	292	41.10	High
[31]	The acceptability and side effects of COVID-19 vaccine among healthcare workers in Nigeria: a cross-sectional study	Nigeria	The six geopolitical zones	Cross-sectional	Doctor Nurse, Medical Laboratory, pharmacist, Physiotherapy, CHEW, Ward Orderly/Porter	309	201	35.00	Low

### Narrative Synthesis

Common determinants of vaccine hesitancy included fears of side effects, lack of trust, and safety concerns. Additional reasons cited for vaccine reluctance included beliefs that the vaccine contains harmful substances, concerns over effectiveness, adverse events following immunization, vaccine had not undergone sufficient clinical trial, government ulterior motive, social media influence, fear of biological chips, conspiracy theory, faulty storage, no exposure to COVID-19, health concerns, RNA component of the vaccine, difficulty in vaccination request, and religious belief. These were not amenable to quantitative analysis.

### Safety of the Vaccine

Several studies in the review [9, 11, 14, 26, 29, 30, 44] revealed that many HCWs were hesitant to accept the newly introduced COVID-19 vaccine due to uncertainties about its safety, which became a major barrier to vaccination uptake. Some of these studies did not report the proportion of participants whose reasons for refusal bordered on safety concerns, but it was common knowledge that the general population dreaded vaccination due to safety concerns.

### Side Effects of the Vaccine

An essential characteristic influencing vaccine acceptance is the side effect profile. Five studies [13, 30, 36, 37, 44] mentioned this outcome, but the actual proportion was not reported. Other studies have also documented that HCWs were deterred from accepting COVID-19 vaccination because of untoward effects [45, 46].

### Social Media Influence and Conspiracy Theory

Negative social media reports and beliefs that vaccines were manufactured to wipe out Africans, were a premise for the non-acceptance of COVID-19 vaccination among HCWs [13, 14]. Conspiracy theories, including government ulterior motives, AstraZeneca not being genuine in Nigeria, fear of biological chips, and fears of vaccines containing dangerous substances are known determinants of vaccine hesitancy among the Nigerian health workforce [14, 39].

### Additional Factors

Vaccine hesitancy among Nigerian HCWs is linked to several other factors. These factors include lack of exposure to the COVID-19 virus, fear of unknown origin, lack of effectiveness, adverse events following immunisation, belief that the vaccine had not undergone sufficient clinical trials and the MRA component of AstraZeneca [14]. In a 2022 study by Emmanuel et al., health concerns, such as blood clots in women (21%), allergic reactions (25%), and innate immunity issues (28%), were identified as significant deterrents to COVID-19 vaccine acceptance among healthcare workers in Nigeria [13].

### Meta-Analysis Findings

#### Pooled Prevalence of Vaccine Hesitancy Among Healthcare Workers in Nigeria

**FIGURE 2:**
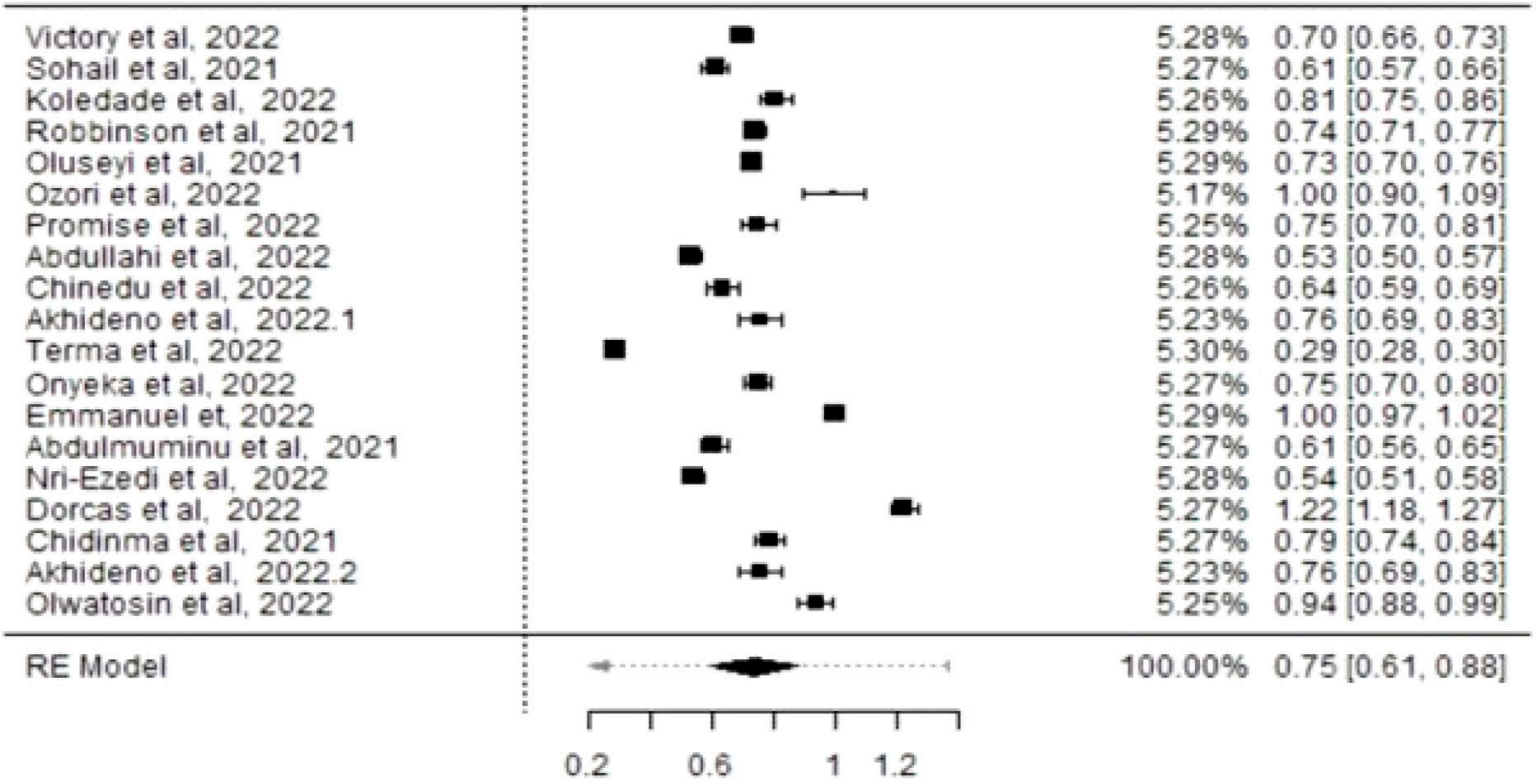
Forest plot corresponding to the pooled prevalence of COVID-19 vaccine hesitancy among Healthcare Workers in Nigeria (Nigeria, 2021/2022).

**FIGURE 3:**
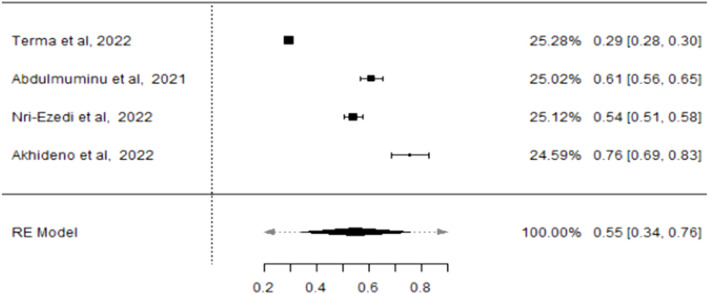
Forest plot showing the prevalence of COVID-19 vaccine hesitancy from subgroup analysis of four studies that recruited medical doctors in Nigeria (Nigeria, 2021/2022).

**FIGURE 4:**
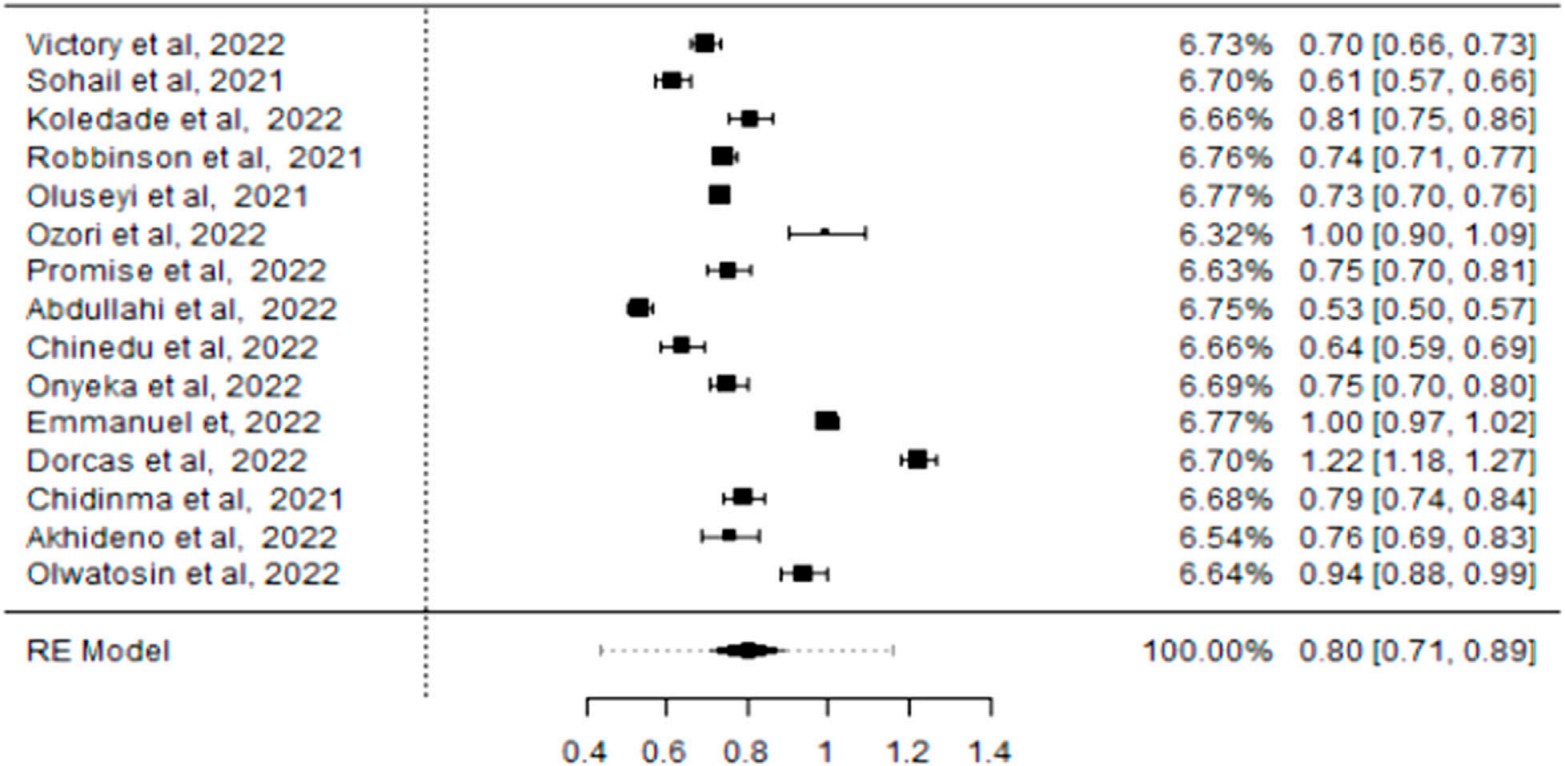
Forest plot showing the pooled prevalence of COVID-19 Vaccine hesitancy following re-analysis (Nigeria 2021/2022).

**FIGURE 5:**
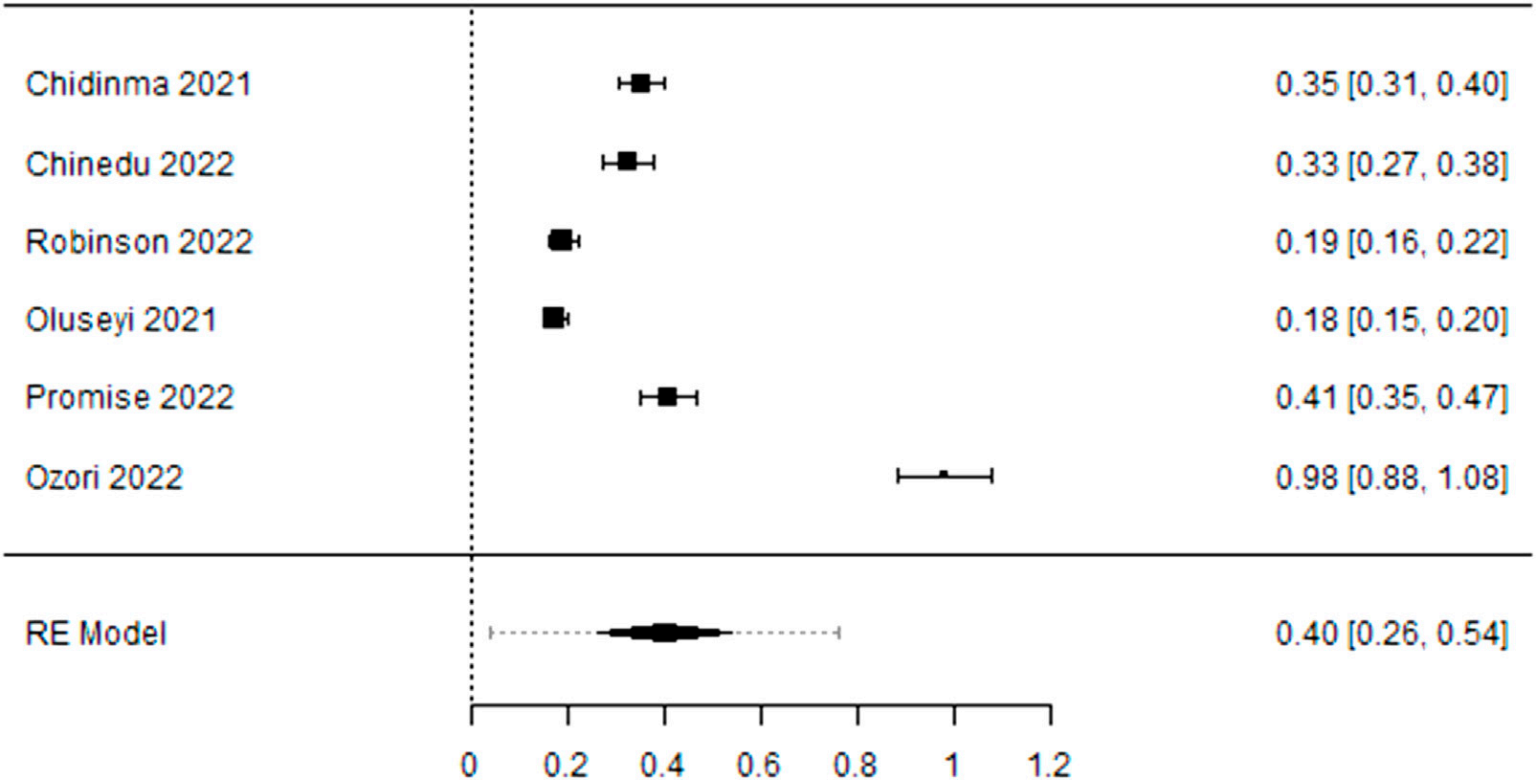
Forest plot showing prevalence of COVID-19 Vaccine hesitancy from subgroup analysis of studies conducted in the Niger Delta Region of Nigeria (Nigeria, 2021/2022).

Using the Freeman-Tukey double arcsine transformation to stabilise variances [47–49], the meta-analysis, using the DerSimonian and Laird random-effects model, estimated a COVID-19 vaccine hesitancy rate of 75% (CI: 61%–88%) among healthcare providers and evaluated heterogeneity measures. The variability among the studies, as presented by I^2^, was 99.69% with a p-value <0.001. The tau effect size variance (0.307), tau^2^ standard deviation (0.0942), and Q statistic (5,826.092) were statistically significant for an overall effect size of 0.75 across the included studies (Figure 2), for statistical output and forest plot. In order to understand sources of the observed variability, subgroup and sensitivity analysis were conducted. To determine whether type of healthcare workers contributed to the heterogeneity, a subgroup analysis of four studies that recruited medical doctors (Figure 3) was performed and substantial heterogeneity persisted after re-analysis (Figure 4). Furthermore, analysis based on the geographical locations in which the studies were carried out were performed. Studies conducted in Niger Delta region of the country were sub grouped and analysed. The I^2^ statistic was 98.45% with a p-value of <0.001 (Figure 5). Additionally, pooled analysis of three studies that were conducted in the Northern part of the country also showed substantial heterogeneity; I^2^: 96.12%, p-value of <0.001 (Supplementary Material S3). Sensitivity analysis taking into account the risk of bias score was conducted. Three studies with moderate risk of bias on the Newcastle Ottawa scale were analysed and showed a variability of 99.44% and a p-value of <0.001 (Supplementary Material S4).

### Reasons for Rejecting COVID-19 Vaccine

#### Side Effects of the Vaccine

In Supplementary Material S5, the forest plot reveals that among HCWs, COVID-19 vaccine refusal due to side effects had a p-value of <0.001, signifying significant variability across studies. The prevalence of side effect-related hesitancy was 76% (CI: 0.57–0.94) with a meta-analysis effect size of 0.76. Tau = 0.212 and Tau^2^ = 0.0448, indicating statistical significance compared with the overall effect size of 0.76. The random effect (represented by I^2^) was 99.24%, primarily due to the variance in the observed events in these studies.

#### Lack of Trust in Vaccines

In Supplementary Material S6, the forest plot shows that among Nigerian HCWs, COVID-19 vaccine hesitancy due to trust issues had a p-value of <0.001, signifying significant variability across studies. The pooled hesitancy prevalence was 55% (CI: 0.042–0.272) with Tau = 0.137 and standard deviation Tau^2^ = 0.0188, demonstrating statistical significance when compared with the overall effect size of 0.55. The random effect (represented by I^2^) was 97.42%, primarily due to the variance in observed events in the studies.

### Safety Concerns

The forest plot depicting the prevalence of vaccine hesitancy due to safety concerns in the included studies yielded a p-value of <0.001, indicating statistical differences in the effects among the studies. Safety concerns were prevalent at 68% (CI: 0.047–0.89). The effect size variance measure was Tau = 0.213, with (SD: Tau^2^ = 0.0452) when, compared with the overall effect size of 0.68 across the included studies. The variability in the meta-analysis, expressed as I^2^, was 98.59%, primarily due to the variance in the observed events within the studies. See Supplementary Material S7 for the statistics and forest plots.

### Certainty of Evidence

The GRADE framework was applied to assess evidence quality in this systematic review and meta-analysis [50, 51]. Observational studies begin with a low evidence rating, which may be adjusted based on specific criteria. As there was no dose-response relationship but rather observed variability due to confounding, the findings could only be rated down. Hence, we examined these domains to assess the robustness of the evidence: risk of bias, inconsistency, indirectness, imprecision, and publication bias.

Risk of Bias: The review relied mainly on cross-sectional studies, a design prone to bias. The Newcastle–Ottawa Scale used for the assessment showed that, 79% of the studies were of low quality, which may be due to non-representative sampling and lack of control of confounders. The overall risk of bias was rated further down in this domain.

Inconsistency: The pooled prevalence of COVID-19 vaccine hesitancy among healthcare workers showed substantial variability (I^2^ = 99.69%) that remained unchanged after a subgroup analysis. This result may be attributable to differences in study populations, regions, and methods. Hence, the evidence was downgraded by one level.

Indirectness: All studies Included in the review recruited healthcare workers in Nigeria to evaluate COVID-19 vaccine hesitancy, so there were no concerns about indirectness. The evidence was not downgraded in this domain.

Imprecision: The evidence was downgraded in this domain as the confidence interval was wide: point estimate 75% (95% CI: 61%–88%). Additionally, the sample sizes showed wide variation especially in smaller studies, leading to concerns about imprecision.

Publication Bias: Funnel plot analysis and Egger’s test did not reveal significant publication bias. Hence no downgrading was applied in this domain.

### Overall Certainty of Evidence

The overarching certainty of evidence for the prevalence of COVID-19 vaccine hesitancy among healthcare workers in Nigeria was judged as low, as a result of serious concerns about bias, inconsistency, and imprecision. Although the studies were relevant, the high heterogeneity and methodological limitations led to low confidence in the level of evidence. Future high-quality, longitudinal studies are needed to improve the certainty of evidence in this area.

## Discussion

Vaccine hesitancy among HCWs is a significant global concern, particularly during pandemics. Their reluctance to get vaccinated can lead to negative attitudes towards vaccination by others, considering their role as advocates of healthy behaviour and health advisors. We conducted a systematic review and meta-analysis to identify the rate of COVID-19 vaccine refusal among HCWs in Nigeria with, the aim of guiding the development of targeted programmes for improving vaccination rates. In this study, the pooled prevalence of COVID-19 vaccine hesitancy among HCWs in Nigeria was 75%. The analysis used a random-effects model because of the significant variation among the studies (I^2^ = 91.96%, p ≤ 0.001). The significant heterogeneity in this study could have arisen from the conduct of individual studies, potentially influencing the results and interpretation [52].

To ensure a comprehensive interpretation of the analysis results, subgroup analysis was performed; however, the observed substantial heterogeneity persisted. This is due to the varied periods, data collection instruments, and potential inconsistencies in the baseline data. In this review, differences in approved vaccine schedules, availability, measurement tools, and the definition of vaccine hesitancy were considered. Furthermore, the study location, heterogeneous nature of the HCW population, and varying sample sizes in the analysed studies may explain these differences.

The COVID-19 vaccine hesitancy rate among HCWs in our study is in line with previous observations in Saudi Arabia 64.9% [53] and France 76.9% [54]. However, a study in Zambia reported a hesitancy rate of 30% [55]. Additionally, our rate was lower than that of studies in China (86.2%) [56], Germany (91.7%) [57], and Canada (80.9%) [58]. A plausible explanation for this discrepancy is that the initial batches of vaccines were manufactured and first administered to HCWs and the general population in European countries and the Americas, and the reported adverse events could serve as a deterrent to COVID-19 vaccine acceptance.

The vaccine hesitancy rate in this study nearly doubled the rate (46%), reported in a recent systematic review and meta-analysis of 15 studies on the prevalence of COVID-19 vaccine hesitancy among HCWs in Sub-Saharan Africa [59]. The sample size, quality assessment tool, and eligibility criteria used in this review may have contributed to the lower prevalence rate reported, but the authors believe that the findings from this review reflects the position of HCWs in Nigeria. Within the African sub-region, higher rates of COVID-19 vaccine hesitancy have been reported in observational studies [60–62]. Diverse reasons for hesitancy towards accepting the COVID-19 vaccine were stated, aligning with factors and predictors observed globally [53, 54, 56–64]. The main reasons were fear of side effects and a lack of trust and safety in the vaccines. Globally, the rates of vaccine hesitancy factors vary. In our study, 76% of HCWs expressed hesitancy because of concerns about the side effects of COVID-19 vaccines. This finding aligns with systematic reviews by Roy et al. (2022) [63] and Wang et al. (2021) [64], which reported 38.73% and three-fold higher odds of vaccine refusal among HCWs linked to side effect concerns. Trust in vaccine safety and, effectiveness is crucial [65,66]. In our study, 55% of HCWs cited lack of trust as a reason for non-acceptance. This has been mentioned in several studies around the world as a key factor implicated in the non-acceptance of COVID-19 vaccines [67–69].

Safety concerns were prominent among Nigerian HCWs that were hesitant about vaccination, with 68% of them expressing such reservations. The rapid approval of COVID-19 vaccines during the evolution of evidence on their effectiveness has contributed to scepticism among both HCWs and the public. The findings from our study align with those of a systematic review of COVID-19 vaccine hesitancy among health workers in America, Asia, Europe, and Africa, where a lack of confidence, safety, and effectiveness regarding vaccines has been reported [69–74]. Other deterrents to COVID-19 vaccine acceptance among HCWs in Nigeria included fear of unknown origin, doubts about effectiveness, concerns about adverse events following immunisation, scepticism regarding sufficient clinical trials, and reservations about the messenger Ribonucleic Acid component of AstraZeneca vaccines [14, 75, 76]. Health concerns, such as reported cases of blood clots after vaccination and allergic reactions, also contributed to hesitancy [13]. Extensive awareness, including rumours that spread through social media such as Facebook and other networks during the pandemic, influenced the observed hesitancy among the study participants [65, 77]. Conversely, a growing body of research consistently indicates that COVID-19 vaccination is both safe and effective. They significantly lower the risk of infection and help prevent the serious consequences of COVID-19 [66]. The benefits of COVID-19 vaccination far outweigh the risks of uncommon adverse effects [66]. Therefore, corrective measures should aim at addressing the disinformation and factors identified as obstacles to COVID-19 vaccination among healthcare practitioners in Nigeria.

The findings of this study hold relevance not only for healthcare workers in Nigeria but also for those in sub-Saharan Africa and globally [74–76]. A thorough, language-unrestricted literature search yielded recent and locally conducted studies assessing COVID-19 vaccine hesitancy among HCWs in Nigeria. The pooled prevalence rates of COVID-19 vaccine hesitancy and associated factors can be instrumental in planning and implementing measures to enhance vaccine uptake among HCWs. Additionally, during the pandemic, global apprehension and doubt about the COVID-19 vaccine hindered widespread acceptance, which is consistent with our findings [14, 39, 45, 46, 76].

### Conclusion

A thorough literature search was conducted to identify current evidence on COVID-19 vaccine hesitancy rates among HCWs in Nigeria. Findings indicate a high rate of vaccine hesitancy among healthcare providers, with an estimated prevalence of 75% (95% CI: 61%–88%, I^2^ = 99.69%, P < 0.001). Primary reasons for hesitancy include concerns over side effects, distrust in vaccine safety, fear of unknown origins, and perceptions of insufficient clinical testing. These obstacles and myths preventing frontline workers from accepting COVID-19 vaccines need to be urgently addressed to improve vaccine uptake among the general population. Future observational studies should adopt written protocols to minimise variability and ensure comprehensive outcome reporting. A systematic review of longitudinal studies could enhance the evidence base on COVID-19 vaccine hesitancy among HCWs.

### Limitations

This is the first systematic review and meta-analysis of COVID-19 vaccine hesitancy among Nigerian HCWs. However, it has some limitations. First, the substantial heterogeneity observed in this meta-analysis (I^2^ = 99.69%) highlights the diverse nature of the included studies. Differences in study settings, healthcare worker categories, data collection approaches and the timing of vaccine rollouts likely contributed to this variability. Despite subgroup analyses and sensitivity testing, significant residual heterogeneity persisted, suggesting unmeasured variations in study methodology.

This review primarily included cross-sectional studies, limiting causal inferences between healthcare providers’ characteristics and vaccine hesitancy [78]. Additionally, none of the included cross-sectional studies offered a protocol that would have helped to guarantee the validity of the findings.

Thirdly, many studies were rated as high risk of bias, potentially inflating the reported prevalence of hesitancy. Publication bias, as indicated by funnel plot asymmetry, may also have influenced our results. This could have skewed the pooled estimate of vaccine hesitancy.

Furthermore, the confidence intervals in several studies were wide, reflecting imprecision in the estimates of vaccine hesitancy. This imprecision was especially pronounced in studies with small sample sizes, contributing to uncertainty around the true prevalence of hesitancy.

This review is limited in its generalizability beyond the Nigerian healthcare context. Furthermore, the heterogeneity in healthcare worker roles included in the studies (e.g., doctors, nurses, pharmacists) suggests that the findings may not be universally applicable to all healthcare professionals.

Throughout the pandemic, people have expressed their opinions and concerns about the vaccine. Thus, eliciting patient perspectives from qualitative evidence may be worthwhile. Despite these limitations, the authors argue that heterogeneity is common in meta-analyses of prevalence studies and should not be used to judge the quality of evidence. Another area for improvement while conducting this systematic review is the need for more access to relevant databases. Although subscription-based databases were not searched, the authors believe that all relevant articles were discovered during the review period, as manual searches were also conducted.

### Amendment of the Study Protocol

This systematic review followed the PRISMA guidelines, but a few deviations were noted from the published protocol [24]. The Web of Science and Embase databases were not searched, and a different statistical package (JAMOVI software) was used for statistical analysis instead of Review Manager Version 5.4. The RevMan software was designed for intervention studies, but a meta-analysis of prevalence studies was conducted.

### Recommendation

This systematic review and meta-analysis relied on secondary data from cross-sectional studies across Nigeria. To enhance quality, future prevalence studies should adhere to standardized protocols and methodological rigor. A heterogeneous healthcare population was recruited from various study centres of which all are not frontline workers. Given the high morbidity and mortality rates associated with the COVID-19 pandemic and the, crucial role of physicians and nurses in flattening the curve, knowing the proportion of physicians and nurses who are hesitant about vaccines would be useful in planning and implementing strategies to increase their uptake. A mixed-method systematic review and meta-analysis is required to address the various myths surrounding vaccine acceptance.
