# Supplementary material for: Prevalence of COVID-19 Vaccine Hesitancy Among Healthcare Workers in Nigeria: A Systematic Review and Meta-Analysis
Source: Int J Public Health. 2025 Feb 5;70:1607655. doi: 10.3389/ijph.2025.1607655 (PMC11836585; doi:10.3389/ijph.2025.1607655)
Supplement: Supplementary file 1 [file Table1.pdf]

Supplementary File. Quality assessment of cross-sectional studies reviewed in 2021/2022 and included in the meta-analysis (Nigeria, 2021/2022)

First author, Year	Representativeness of the sample	Sample size	Non-respondent	Ascertainment of exposure status (risk facto)	Comparability	Assessment of the outcome	Statistical test	Total score
Terma et al. (2022) [26]	*	-	-	*	**	-	*	5stars
Emmanuel et al. (2022) [27]	-	-	*	**	-	-	*	3stars
Onyeka et al. (2021) [28]	*	-	-	-	**	-	*	4stars
Emmanuel et al. (2022) [29]	-	-	-	-	-	-	*	1star
Abdulmuminu et al. (2021) [30]	*	-	-	-	-	-	*	2stars
Nri-Ezedi et al. (2022) [31]	*	*	*	-	**	-	*	5stars
Dorcas et al. (2022) [32]	-	-	-	*	**	-	-	3stars
Chidinma et al. (2021) [33]	*	-	-	-	**	-	*	4stars

First author, Year	Representativeness of the sample	Sample size	Non-respondent	Ascertainment of exposure status (risk facto)	Comparability	Assessment of the outcome	Statistical test	Total score
Akhideno et al. (2022) [34]	*	-	-	-	**	-	*	4stars
Chinedu et.al, 2022 [35]	-	*	-	-	**	-	*	4stars
Abdullahi et al. (2022) [36]	*	-	-	-	**	-	*	4stars
Promise et al. (2022) [37]	-	-	-	-	**	-	*	3stars
Ozori et al. (2022) [38]	*	*	-	-	**	-	*	5stars
Oluseyi et al. (2021) [39]	-	-	-	-	**	-	*	3stars
Robbinson et al. (2022) [40]	-	-	-	-	**	-	-	2stars
Koledade et al. (2022) [41]	-	-	-	-	-	-	*	1star
Sohail et al. (2021) [42]	-	-	-	-	-	-	*	1star
Victory et al. (2022) [43]	-	-	-	-	**	-	*	3stars

First author, Year	Representativeness of the sample	Sample size	Non-respondent	Ascertainment of exposure status (risk facto)	Comparability	Assessment of the outcome	Statistical test	Total score
Oluwatosin et al. (2021) [44]	**	**	-	-	*	**	**	9stars

Foot Notes: ** Quality criterion completely satisfied; *quality criterion satisfied; - quality criterion not satisfied or insufficient information to label as satisfied.
