# Supplementary material for: Prevalence of COVID-19 Vaccine Hesitancy Among Healthcare Workers in Nigeria: A Systematic Review and Meta-Analysis
Source: Int J Public Health. 2025 Feb 5;70:1607655. doi: 10.3389/ijph.2025.1607655 (PMC11836585; doi:10.3389/ijph.2025.1607655)
Supplement: Supplementary file 2 [file DataSheet1.pdf]

Random-Effects Model (k = 3)

	Estimate	se	Z	p	CI Lower Bound	CI Upper Bound
Intercept	0.307	0.0578	5.31	< .001	0.194	0.421
	.	.	.	.	.	.

Note. Tau² Estimator: DerSimonian-Laird

Heterogeneity Statistics

Tau	Tau²	I^2	H^2	R^2	df	Q	p
0.098	0.0096 (SE= 0.0105)	96.12%	25.770	.	2.000	51.540	< .001

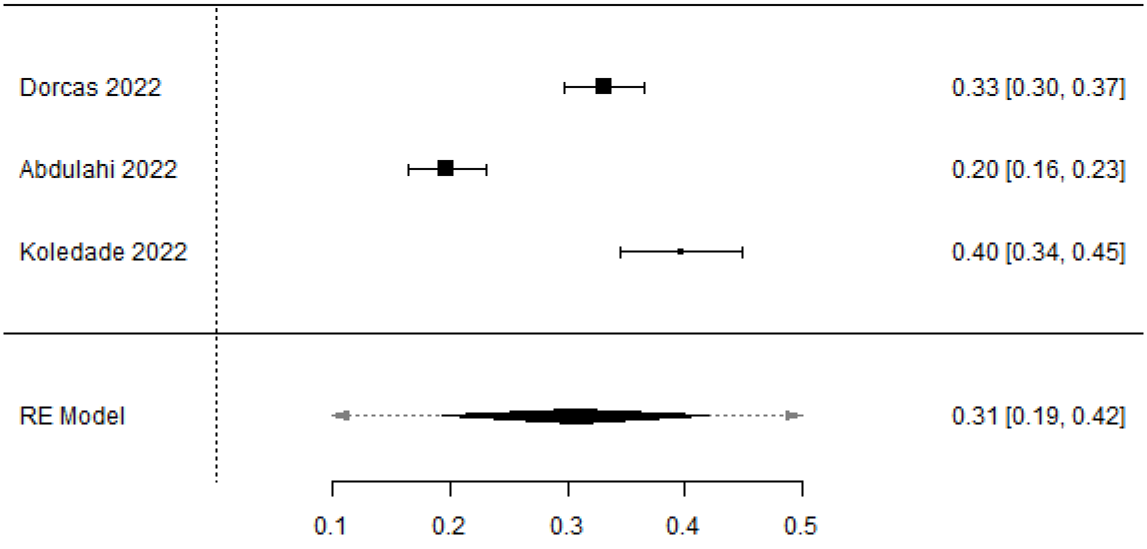

Supplementary File 2.

Random-Effects Model (k = 3)

	**Estimate**	**se**	**Z**	**p**	**CI Lower Bound**	**CI Upper Bound**
Intercept	0.284	0.125	2.27	0.023	0.038	0.530
	.	.	.	.	.	.

Note. Tau² Estimator: DerSimonian-Laird

Heterogeneity Statistics

**Tau**	**Tau²**	**I²**	**H²**	**R^2**	**df**	**Q**	**p**
0.216	0.0467 (SE= 0.0576)	99.44%	177.303	.	2.000	354.606	< .001

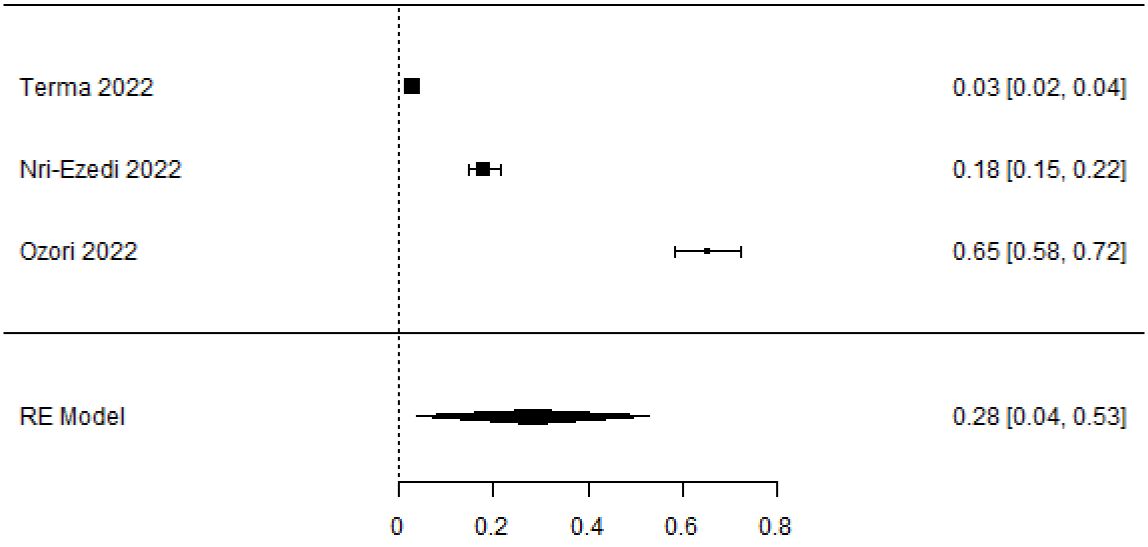

Supplementary File 3.

Random-Effects Model (k = 5)

	Estimate	SE	Z	p	CI Lower Bound	CI Upper Bound
Intercept	0.757	0.0951	7.96	< .001	0.571	0.944
	.	.	.	.	.	.

Note. Tau² Estimator: DerSimonian-Laird

Heterogeneity Statistics

Tau	Tau²	I²	H²	R²	df	Q	p
0.212	0.0448 (SE= 0.0348)	99.24%	130.736	.	4.000	522.945	< .001

Heterogeneity Statistics

Tau	Tau²	I^2	H^2	R^2	df	Q	p

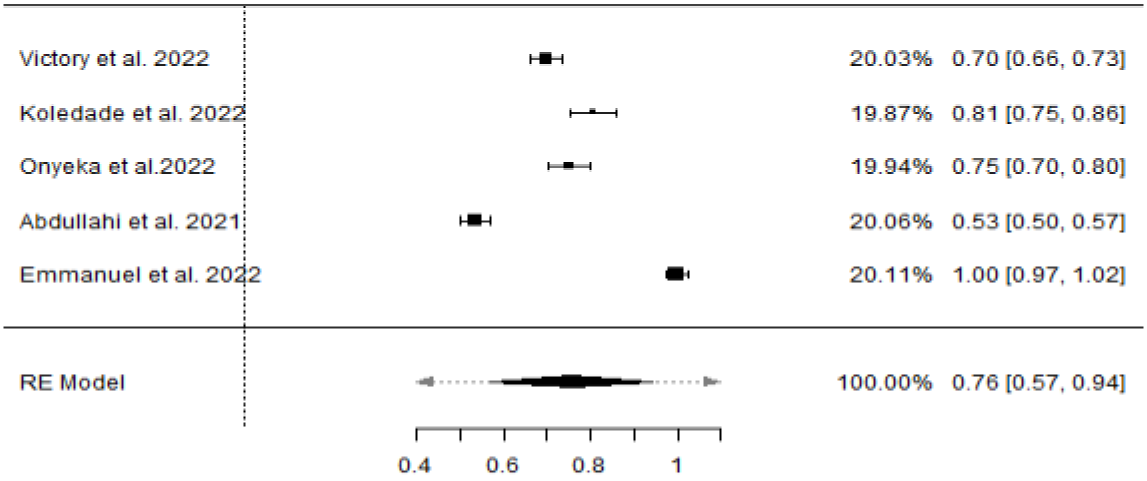

Supplementary File 4.

Random-Effects Model (k = 4)

	Estimate	SE	Z	p	CI Lower Bound	CI Upper Bound
Intercept	0.552	0.0696	7.92	< .001	0.415	0.688
	.	.	.	.	.	.

Random-Effects Model (k = 4)

	**Estimate**	**SE**	**Z**	**p**	**CI Lower Bound**	**CI Upper Bound**

Note. Tau^2 Estimator: DerSimonian-Laird

Heterogeneity Statistics

**Tau**	**Tau^2**	**I^2**	**H^2**	**R^2**	**df**	**Q**	**p**
0.137	0.0188 (SE= 0.0167)	97.42%	38.704	.	3.000	116.111	< .001

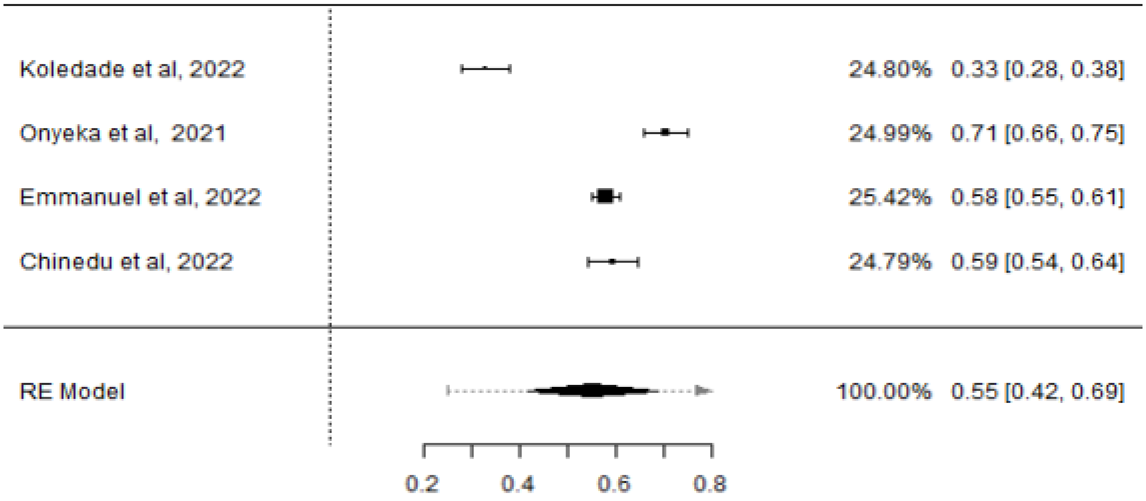

Supplementary File 5.

Random-Effects Model (k = 4)

	**Estimate**	**SE**	**Z**	**p**	**CI Lower Bound**	**CI Upper Bound**

Random-Effects Model (k = 4)

	Estimate	SE	Z	p	CI Lower Bound	CI Upper Bound
Intercept	0.680	0.107	6.35	< .001	0.470	0.890
	.	.	.	.	.	.

Note. Tau^2 Estimator: DerSimonian-Laird

Heterogeneity Statistics

**Tau**	**Tau^2**	**I^2**	**H^2**	**R^2**	**df**	**Q**	**p**
0.213	0.0452 (SE= 0.0399)	98.59%	70.709	.	3.000	212.127	< .001

Random-Effects Model (k = 4)

	Estimate	SE	Z	p	CI Lower Bound	CI Upper Bound

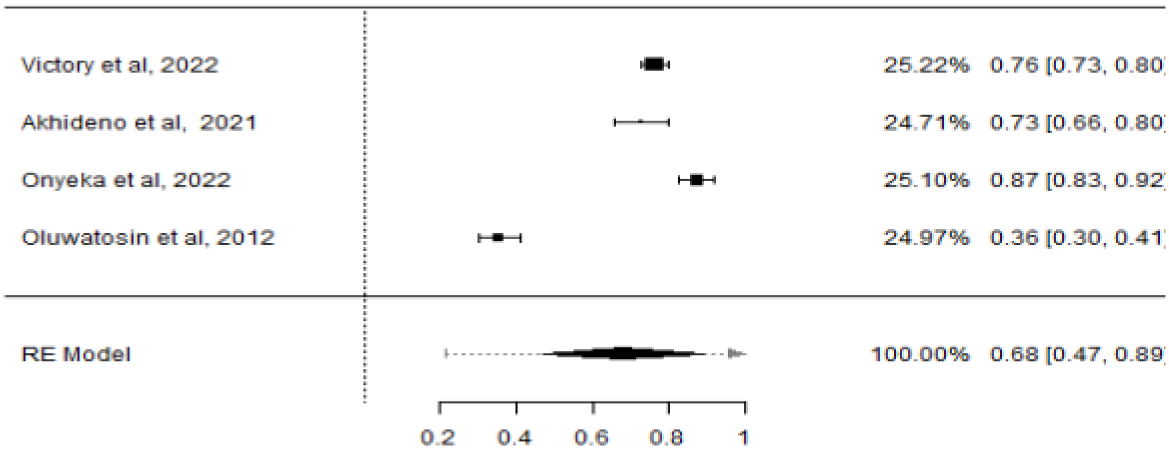

Supplementary File 6.
